# Benign growing mass of the digit presenting as an ulcerated mass – case report and review of the literature

**DOI:** 10.1080/23320885.2021.1962718

**Published:** 2021-08-13

**Authors:** J. Nunes Pombo, A. Nixon Martins, C. Paias Gouveia, B. Pena, D. López-Presa, G. Ribeiro

**Affiliations:** a Plastic, Reconstructive and Aesthetic Surgery, Hospital de Santa Maria (Centro Hospitalar Lisboa Norte), Lisbon, Portugal; b Anatomic Pathology, Hospital de Santa Maria (Centro Hospitalar Lisboa Norte), Lisbon, Portugal

**Keywords:** Fibro-osseous pseudotumor digit, hand tumor, hand surgery

## Abstract

A 68 year-old female presents with an ulcerated mass of the 5th digit, with rapid growth during the previous month to surgery. The mass was excised and covered with a 4th dorsal metacarpal artery perforator flap. The histologic analysis was compatible with the diagnosis of fibro-osseous pseudotumor of the digit.

## Introduction

The hand surgeon acts both as an oncological surgeon, ablating all the tumor mass, potentially compromising aesthetics and function, and as a reconstructive surgeon, trying to optimize hand function. Balancing these goals may be a demanding task [1].

Even though the majority of hand tumors are benign, especially if they do not involve the skin, some malignant tumors arise in the hand. In the latter, a more aggressive approach would be justified [2].

Fibro-osseous Pseudotumor of the Digits (FOPD) is a rare benign tumor with clinical characteristics that can mimic a malignant tumor. We present a case of FOPD and a review of the literature.

## Case description

**Figure 1.**
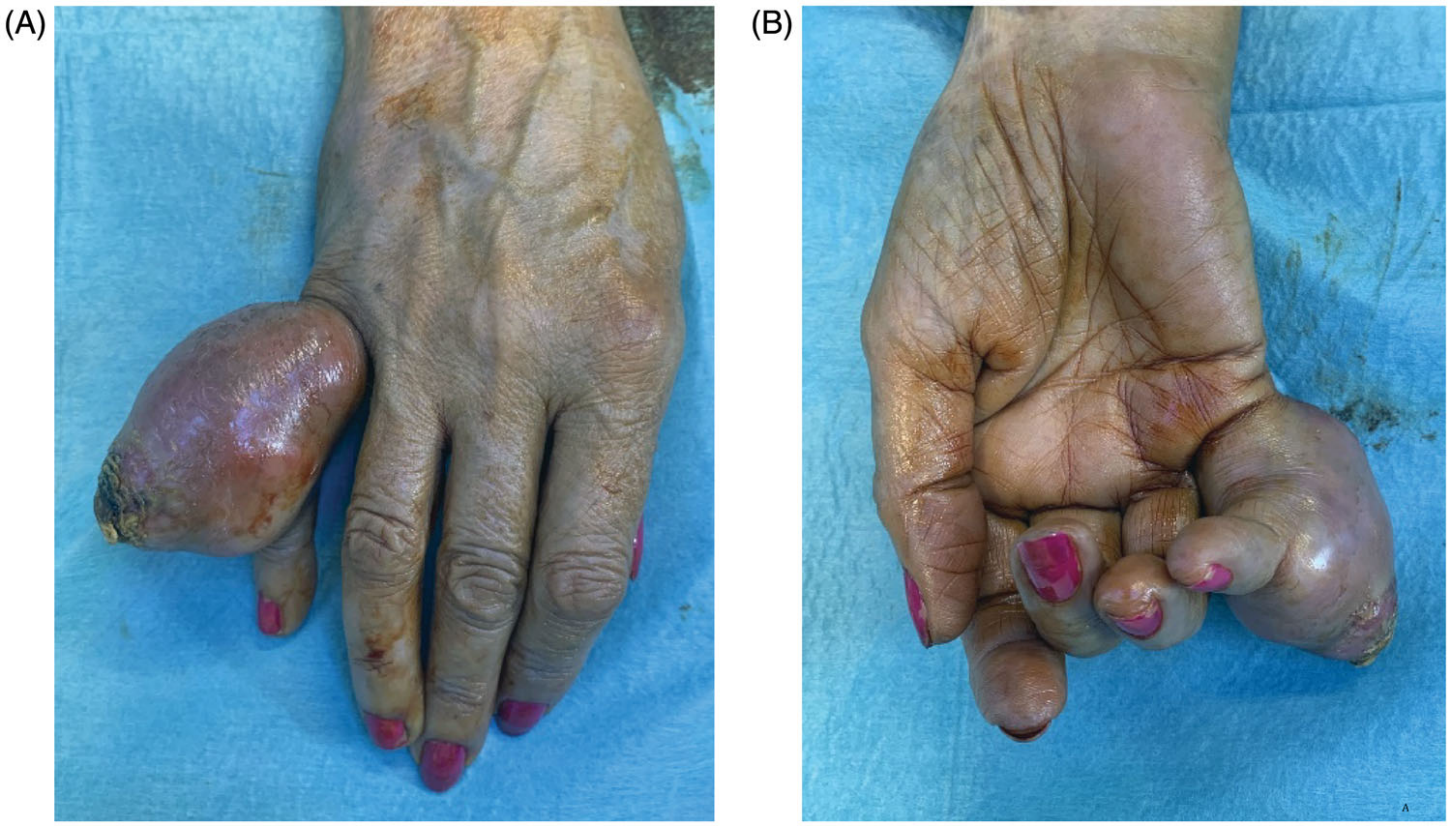
Preoperative photographs of the tumor, dorsal view (A), palmar view (B).

A 68 year-old female, smoker, presented to the ER with an ulcerated mass of the dorsum of the 5th finger involving the proximal phalanx to the DIP joint (Figure 1). It had been progressively enlarging for the past year with substantial growth in the previous month. It was presented as a painless mass that limited PIP flexion. The neurovascular examination was normal. The ultrasound showed a 4.4 × 2.6 cm hypoechogenic mass with a significant doppler sign.

**Figure 2.**
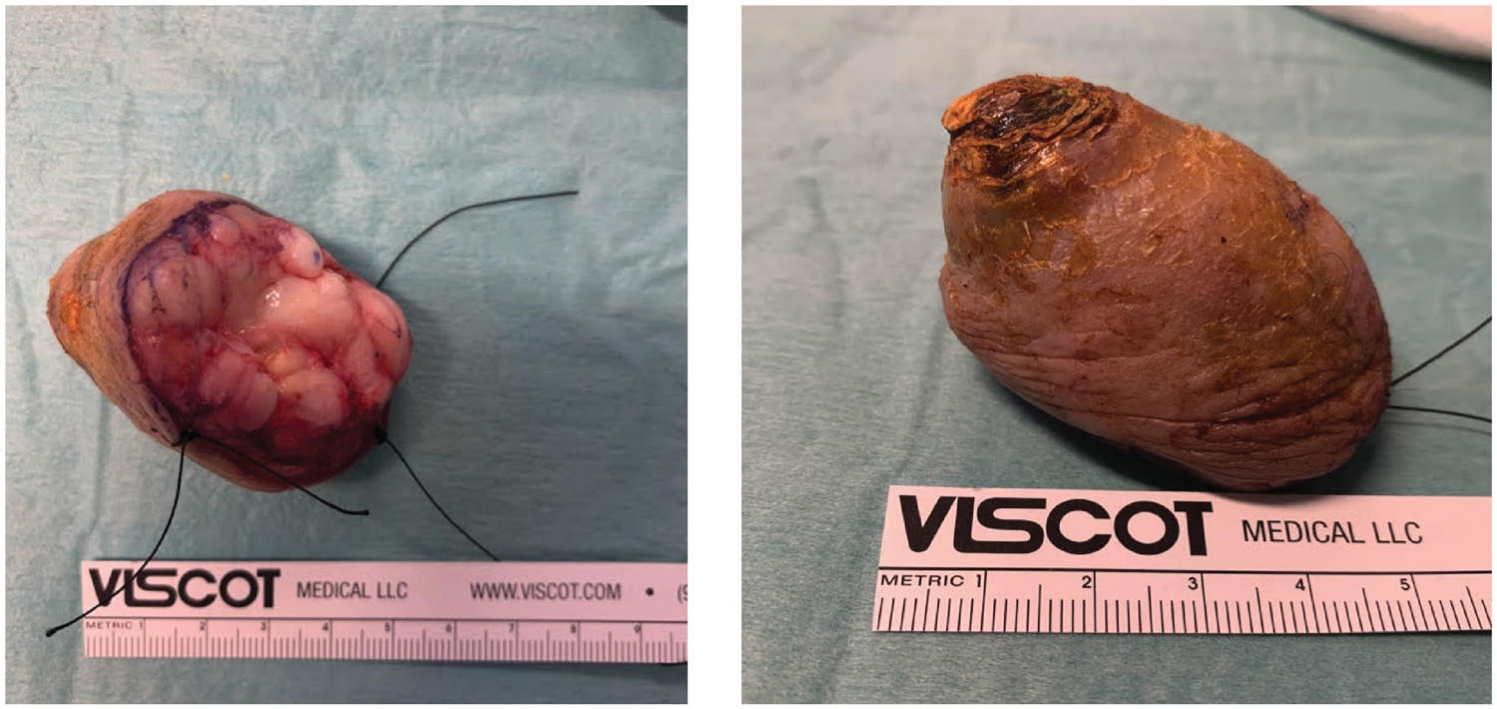
Post-excisional photographs of tumor: inferior (A) and lateral (B) views.

**Figure 3.**
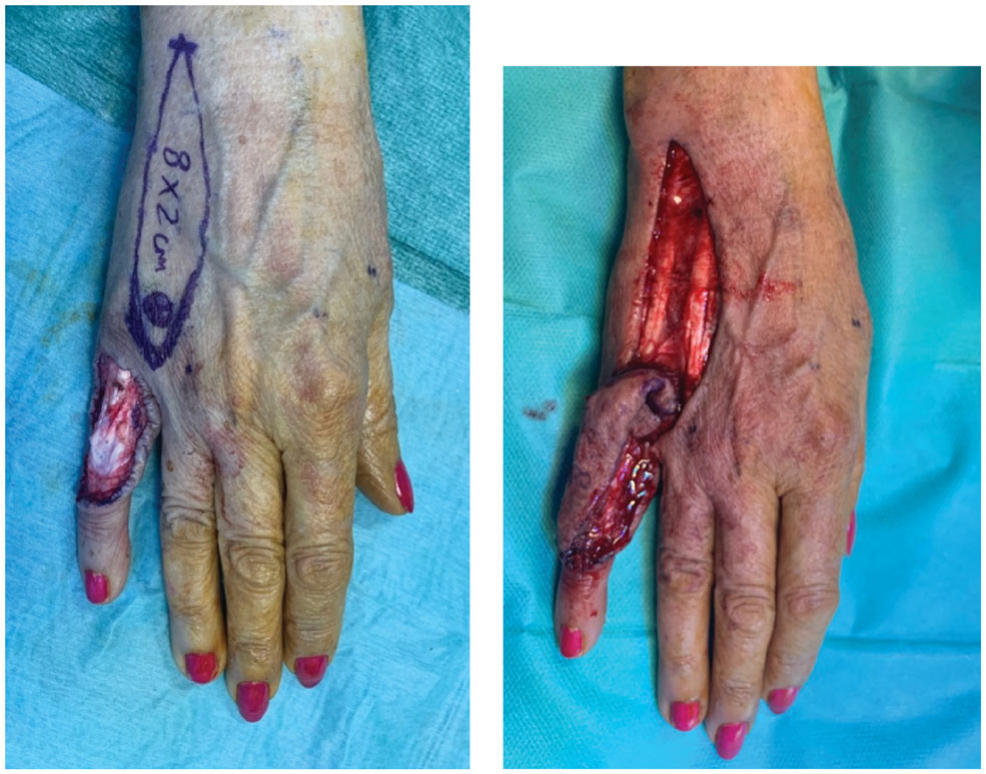
Intra-operative views after surgical excision of the tumor with preservation of the extensor apparatus (A) and after raising the flap (B).

**Figure 4.**
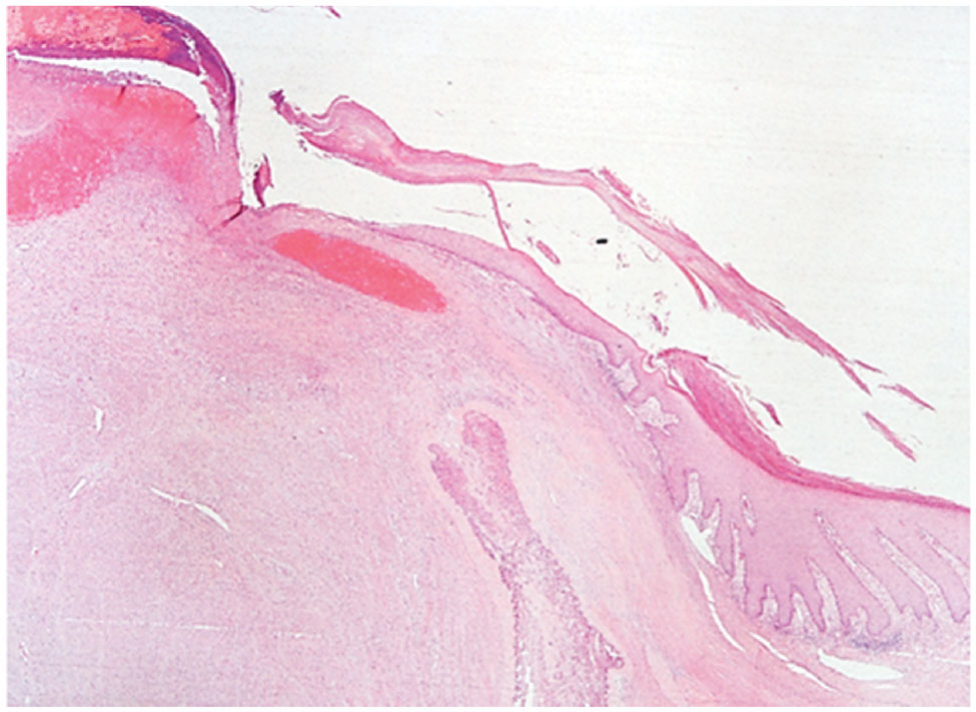
Fibro-osseous pseudotumor of digits. The lesion presents as a well-circumscribed dermal mass, with epidermal ulceration. Hematoxylin-eosin × 25.

**Figure 5.**
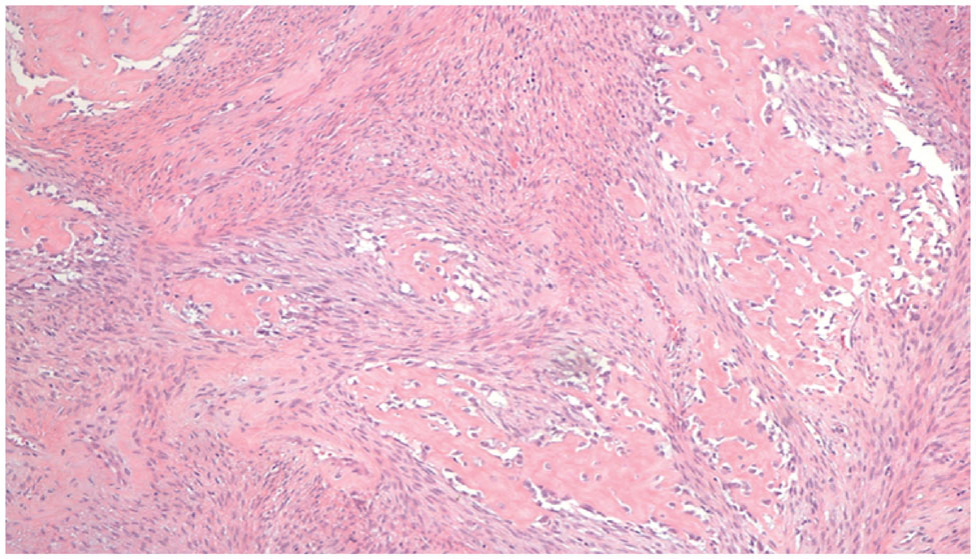
Fibro-osseous pseudotumor of digits. At medium power, the mixture of arranged fibroblasts and myofibroblasts and the deposits of osteoid is rimmed by uniform osteoblasts. Hematoxylin-eosin × 100.

**Figure 6.**
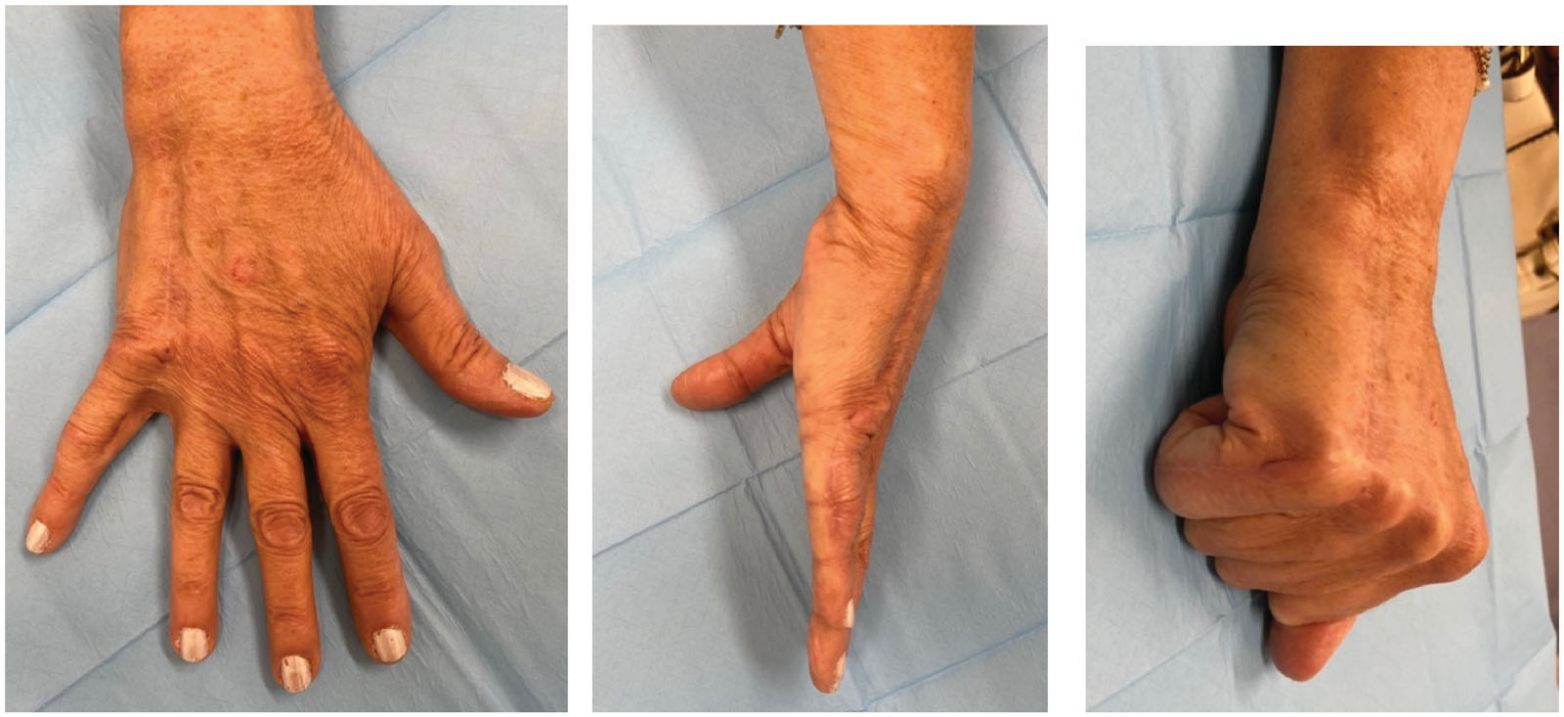
Post-operative views at 22 months showing complete wound healing without functional limitation: dorsal (A), and medial (B,C) views.

Excision with the overlying skin was performed down to a tumor less surgical plane, preserving the extensor apparatus (Figure 2). The resulting defect was covered with a 4th dorsal metacarpal artery perforator flap and the donor area primarily closed (Figure 3). Histopathological study revealed a lobulated tumor, self-limited and centered in the dermis, causing epidermal ulceration. Histologically, it is composed of fascicles of uniform spindle cells, admixed woven bone without zonation. There’s a mixture of fibroblasts and myofibroblasts, arranged in hyper and hypocellular hyalinized areas and deposits of osteoid rimmed by uniform osteoblasts. Cells have bland cytology and no necrosis or mitotic figures are seen, compatible with the diagnosis of FOPD (Figure 4 and 5). The patient was followed for 22 months with no evidence of recurrence (Figure 6).

## Discussion

FOPD is a rare entity. There are 173 cases documented in the literature [3–54], totaling 174 with the present case report (Table 1). The first case dates back to 1931. There have been several terms to classify this entity including: ‘pseudo-malignant osseous tumor of soft tissue’ [36], ‘parosteal fasciitis’ [49], and ‘florid reactive periostitis’ [3,4,7,13]. This disease was unified under the term ‘fibro-osseous pseudotumor of the digits’ in 1986 by Dupree and Enzinger [50]. The tumor is characterized by reactive fibroblastic proliferation and focal bone formation limited to the skin and subcutaneous tissue. The tumor is not of bone origin, though [52]. It presents as an enlarging mass, which can be tender or painless. Even though a history of trauma has been described in as many as 40% of patients, it is hard to assess the relevance and prevalence of injury since minor trauma of the extremities is common. Skin ulceration may occur; the tumor mimics pyogenic granuloma, especially if it affects the toes [7,31,38,48]. Due to its growth and local aggressiveness, it can simulate malignancies. When mentioned, we found a high rate of suspicion of sarcoma [14,19,50,51], namely osteogenic sarcoma. Some case series did not report the individual patient data regarding age [19,46,50]. The age ranged from 5 to 81, with a mean age of 36 and a median of 30 years old. The majority of patients were female (56.9%, 99 cases). It is more frequent in the upper limb (82.6%) than in the lower limb (16.9%). There is a sole case of FOPD outside the extremities [28]. In the upper limb, the most common segment affected is the finger (76.8%), more precisely the 2nd finger (32%) and the proximal phalanx (62.3%), followed by the hand (21.1%) and wrist (2.1%).

**Table 1.** FOPD reported cases in the literature.

References	Reported cases	Age (years)	Gender	Patient history	Clinical presentation	Anatomical location	Ulceration	Treatment	Recurrence	Follow up
Mallory [3]	1	17	F	–	Wart-like lesion for 3 years	3rd intermetacarpal space right hand	–	Local excision	–	–
Gonçalves [4]	1	23	F	–	Painful swelling	P1D2 left hand	–	Transmetacarpal amputation	–	60 months
McCarthy et al. [5]	1	40	M	–	Growing mass, 6 months	P1D4 right hand	–	Ray amputation	–	12 months
Spjut and Dorfman [6]	12	5–46 (mean 22.8, median 19)	5 M, 7 F	–	Swelling mass (12), pain (5) and redness (3)	Hand: D3 (4); D4 (3); D5 (2); Foot: D1 (2). Hand: P1 (6); P2 (3), MC (1); Foot: P3 (1); MT (1)	–	Local excision (11), ray amputation (1)	1 Recurrence 10 months after local excision.	2 months–11 years
De Smet and Vercauteren [7]	1	58	F	–	Painful swelling mass with, impaired flexion of joint	P1D3 left hand	–	Transmetacarpal amputation	–	12 months
Bulstrode et al. [8]	1	30	F	–	Stiffness and weakness, 2 years + tender nodule, 2 weeks	Left hand	–	Local excision followed by transmetacarpical amputation of 3 digits	Recurrence 2 months after associated with refractory pain, no recurrence after amputation	12 months
Patel and Desai [9]	1	35	F	–	Painful growing mass, 1 year	P1D5 left hand	–	Local excision followed by ray amputation	Recurrence after local excision. no recurrence after amputation.	8 years
Dupree and Enzinger [10]	21	8–70 (median 33)	13 F 8 M	History of trauma injury site	Sudden (9)/slowly (3) growing mass; Painful (12), erythematous (11); hematoma (1), cellulitis (5)	DD1 (3); D2 (6); D3 (5); D4 (3); D5 (4). P1 (6); P2 (1); P3 (3); not stated (11)	–	Amputation(2), radical excision (1), Local Excision (18)	Recurrence 1 case 1 year after incomplete excision. no recurrence after amputation	Follow up in 7 cases, up to 15 years
Chan et al. [11]	1	17	M	History of trauma injury site	Enlarging polypoid nodule 2 months	P3D2 left foot	Present	Local excision ×2, followed by amputation by DIP joint	Recurrence after 2 local excision. no recurrence after amputation.	12 months
Franchi et al. [12]	1	55	M	–	Painless mass for 3 years	P1D3 right hand	–	Local excision	–	24 months
Aboujaoude et al. [13]	1	13	F	–	–	Left hand	–	Local excision	–	–
Horie and Morimura [14]	1	59	F	–	Painful polypoid mass, 1 month, pyogenic granuloma-like	P2D1 right foot, subungual	Present	Local excision	–	4 months
Prevel and Hanel [15]	1	25	F	–	Growing mass, 6 months	P3D2 right hand, ungual	–	Local excision ×2	Recurrence 10 months after excision	24 months
Riaz et al. [16]	1	48	M	–	Swelling mass associated with pain and stiffness	P1D1 left hand	–	Local excision	–	6 months
Tang et al. [17]	1	53	F	–	Painful swelling	M4 left hand	–	Local excision	–	–
Sleater et al. [18]	3	47–81 (mean 61.3333)	2 M 1 F	–	–	D2 (1), D2 foot (1), P3D3 (1)	1 out of 3	–	–	–
Moon et al. [19]	1	10	M	–	Growing mass 4 months with hyperkeratotic cap	Subungual P3 hallux, right foot	–	Local excision	–	9 months
Takahashi et al. [20]	1	60	F	–	Painless swelling for 3 months	P3D2 right hand, subungual	–	–	–	–
Nishio et al. [21]	1	30	F	–	Painful swelling with rapid increase over 4 weeks and restriction of PIP joint	P2D4 left hand	–	Local excision	–	7 months
Solana et al. [22]	1	56	M	–	Tender growing mass, 3 months	P1D1 left hand	–	Local excision	–	24 months
de Silva and Reid [23]	14	17–75 (mean 38.1, median 35.5)	7 M 7 F	–	Painful growing mass 75%, mean duration 9.7 weeks	D1 (2), D2 (4), D3 (5), D5 (3)	–	(4) Biopsy, (9) Excision, (1) finger amputation *initial misdiagnosed as osteosarcoma	Local recurrence 24 months after excision (1)	–
Hirao et al. [24]	1	29	F	–	Swelling and painful mass, pyogenic granuloma-like	1st Intermetatarsal space, left foot	–	Local excision	–	17 months
Coleman [25]	1	70	F	–	Growing nodule for the past 2 years with rapid growth over 2 months	P3D4 left hand	–	Ray amputation	–	10 months
Usta et al. [26]	1	57	M	–	Painful swelling 2 years	P1D2 left hand	–	Local excision	–	–
Moosavi et al. [27]	43	10–64 (40 mean; 40 median)	17 M 26 F	History of trauma (6) + occupation with continuous hand-work	Pain and edema	Hand (6), Finger (1), D1 hand (3), D2 hand (11), D3 (10), D4 hand (4), D5 hand (2), hypothenar eminence (1), web space (2), wrist (1), foot (1), D1 foot (1)	6 out of 7	Local excision (42), ray amputation, misdiagnosed as malignancy (1)	–	–
Nalbantoglu et al. [28]	1	30	M	–	Pain and swelling on the radial side of the right wrist, 4 months	Wrist: 1 cm distal to the radial styloid over the first dorsal-extensor compartment	–	Local excision	–	24 months
Kaddoura and Zaatari [29]	1	42	M	–	Erythematous nodule with rapid growth over 2 months and progressively painful, limiting flexion and abduction of thumb	Thenar eminence and 1st interdigital space, left hand	–	Local excision	–	18 months
Bettex et al. [30]	1	15	M	–	Slow-growing mass	D1	–	Local excision	–	15 months
Chaudhry et al. [31]	17	5–64 (median 34)	8 M 9 F	History of trauma on injury site (2)	Nodule (12), swelling (3), verrucous lesion (1), purulent lesion (1), duration from 2 weeks to 2 years	Ungual D1 foot (1), Ungual D2 foot (1), Ungual D4 foot (1), D4 foot (1), D5 foot (1) D1 foot (2), D2 hand (5), D3 hand (2), D5 hand (1), foot (1), forehead (1)	7 out of 17	Local excision (16), Finger amputation (1) * recurrence with later diagnosis of sarcoma	Local recurrence (2)	14 cases: 12–168 months. Median 45 months, average 62 months
Song et al. [32]	1	20	F	–	Painful mass	P1D1 left foot	–	Local excision	–	–
Tan et al. [33]	1	54	F	–	Nodule mimicking pyogenic granuloma: tender, mobile and erythematous nodule, growing for 2 months	P2D1 left foot	Present	Local excision	–	–
Sayar et al. [34]	1	30	F	History of trauma injury site	Painful swelling mass	P2D5 left foot	–	Local excision	–	24 months
Anand et al. [35]	1	21	F	–	Swelling, 3 months	M4 left hand	–	Local excision	–	6 months
Lee et al. [36]	1	22	F	–	–	D2 left hand	–	Local excision	–	7 months
Seeger et al. [37]	1	5	M	–	Swelling mass	M3 right hand	–	Local excision	–	12 months
Javdan and Tahririan [38]	1	30	M	–	Painful growing mass for 12 months, finger motion slightly limited	P2D2 left hand	–	Local excision	–	–
Cornerc et al. [39]	1	61	F	Rheumatoid Arthritis, treatment with methotrexate	Painless tumefaction with increase over 1 month	P2D2 right hand	–	Amputation	–	–
Hashmi et al. [40]	1	32	M	Rural laborer by occupation	Painless mass for 3 years	D3	Present	Local excision	–	12 months
Kwak et al. [41]	1	19	F	–	Painful growing mass, 12 months	P1D3 right hand	–	Local excision	–	12 months
Zhou et al. [42]	1	48	M	–	Painful erythematous mass worsening 2 months	P2D4	–	Narrow-margin excision with recurrence followed by complete excision	–	6 months
Meani et al. [43]	1	16	M	Crush injury 2 years before	Painful subungual hematoma and swelling of toe, 2 years after trauma	P2D1 left foot	–	Curettage and nail plate removal + local excision	Recurrence 6 weeks after curettage, no recurrence after local excision	12 months
Kontogeorgakos et al. [44]	1	28	F	Hairdresser working with scissors with the hand	Erythematous swelling with progressive pain over 3 months, Tinnel's sign positive	Dorsum of 1st intermetacarpal space, left hand	–	Local excision	–	12 months
Choi et al. [45]	1	68	M	Type 2 DM	Growing tender subcutaneous mass for 3 months	P1D1	–	Local excision	–	6 months
Singal et al. [46]	1	36	F	–	Painless lesion growing for the past 6 months	Subungual P3D2 right hand	–	Local excision	–	8 months
Gómez-Zubiaur et al. [47]	1	13	F	Cuticle manipulation	Mass mimicking pyogenic granuloma: Painful, erythematous mass with granulomatous bleeding surface growing for the previous 2 months	P3D3 left hand periungual	Present	Shaving followed by local excision	–	–
Cho et al. [48]	1	27	M	History of trauma injury site	Painful erythematous mass for 2 months, viral wart like	P3D3 foot	Present	Local excision	–	6 months
Takahashi et al. [49]	1	36	M	–	Subungual painful keratotic nodule, 2 months	Subungual P3D5 left foot	–	Local excision	–	–
Flucke et al. [50]	5	33–72 (mean 48)	2 M 3 F	–	–	Palm (2), D3 (1), thenar eminence (1), D1 foot (1)	–	Local excision (5)	–	–
Jawadi et al. [51]	1	27	F	–	Growing mass for 7 months	P1D4	Present	Local excision	–	–
Švajdler et al. [52]	12	5–64 (median 32)	6 M 6 F	–	Mass (9), granulation tissue (1), painful mass (1), osteochondroma (1)	Wrist (1) , M3 (1), D4 foot (1), D1 foot (2), 1st metacarpal, thenar eminence (1) , digit of the hand (1) D5 foot (1), D5 hand (1), hypothenar eminence (1), hand/metacarpus/digit (1)	–	–	–	–
Sakuda et al. [53]	1	30	M	Suspected occupational injury due to excessive load	Growing mass	D5 right hand	–	Local excision	–	6 months
Rela and Bantik [54]	1	60	F	hypothyroidism on thyroxine replacement; right thumbnail with a ‘vertical split in the midline’	Pain and swelling for 3 weeks	P2D1 right hand, subungual	–	Washout and debridement	–	6 months

M: male; F: female; D: digit; P: phalanx; M: metacarpal.

Radiologically, FOPD usually presents as a soft tissue mass with ill-defined margins and might reveal extraosseous ossification and bone formation. The periosteal reaction has been described [42]. Cortical involvement or bony destruction seldom occurs. MRI findings are commonly non-specific and frequently demonstrate a non-invasive soft tissue mass.

Histologically, FOPD presents as a multi-nodular lesion with irregular margins and it is localized in the dermis and subcutaneous tissues. Muscular and cartilage involvement is absent.

Typically, FOPD shows fibroblastic/myofibroblastic proliferation without atypia, in a myxoid stroma, and immature trabeculae with osteoid rimmed by osteoblasts, without zoning phenomenon [10,23,51]. Osteoclasts and bone marrow elements are rarely seen [42].

Immunohistochemically, the majority of studies has demonstrated positive staining for vimentin 14 [11,21,25,26,34], and focal reactivity to actin [18,21,26,27,40,51,53], which is in favor of myofibroblastic differentiation [18]. There is, however, controversial data, since Chan et al. found no reactivity to actin, myosin, myoglobin, desmin, and no dense bodies on electron micrograph, which is against the involvement of myofibroblasts [11], and Sayar et al. found no evidence of immune-reactivity to actin and desmin [34].

The majority of cases demonstrated negative staining to S – 100, CD 34, cytokeratin (MAK-6 and CAM 5.2), desmin, and epithelial membrane antigen (EMA) [3,11,18,25–27]. Diagnosis is challenging – FOPD is a rare disease with only 174 cases documented, and it shares many histological and clinical features with malignant disease. This might lead to incorrect diagnoses, such as extraskeletal osteosarcoma, resulting in unnecessary procedures, including amputation. It should, however, be considered as a potential diagnosis of fast-growing tumors of the hand or feet with no previous history of trauma [42]. Osteosarcoma is usually diagnosed in older people (scarcely under 40) and is rarely found in the fingers, being more common in the large bones of the upper and lower extremities [42]. Myositis ossificans has been associated with FODP, being the latter considered by some authors as a superficial variant of the first. However, in myositis ossificans there is commonly a history of trauma [38,42,51]. Other differential diagnosis include bizarre parosteal osteochondromatous proliferation (Nora’s lesion), ossifying plexiform tumor, acral osteoma cutis, and subungual exostosis [45,50].

FOPD might be included in a group of USP-6-rearranged myofibroblastic neoplasms that share a genetic rearrangement of the USP-6 gene and are known for their rapid yet self-limited growth and low recurrence rate [52,55]. Recent studies have identified COL1A1-USP6 fusions in the majority of their FOPD cases [50,52,56]. Molecular and genetic studies can therefore help diagnosis and avoid over-treatment, particularly in cases where biopsy is difficult to obtain [52,55], or clinical history is insufficient [52] since these lesions frequently mimic soft tissue sarcomas [52], but are self-limited and can be cured with a more conservative surgical excision [55].

The treatment for FOPD is surgical excision. Prognosis is excellent as recurrence has been related to incomplete excision [10,11,42]. We found 13 cases in which amputation was the treatment of choice [4–11,23,25,27,39]. Some of these cases were initially suspected or diagnosed as malignant – de Silva and Reid described a 13% of malignancy assumption by radiologists and a 9% incorrect malignant diagnosis by pathologists [23], while in others the extension of the disease and bone destruction led the surgeons to decide for amputation.

Nine cases of recurrence were described in the literature [6,8–11,15,23,31,43]. Most of them did not provide information about the reason for the recurrence. However, in three cases incomplete excision seems to be the reason for it thus reinforcing the need for complete excision.

After complete removal of the tumor, no recurrences were found with a median follow up of 15 (4–168) months [4,5,7–16,19,21–25,28–31,34–37,40–46,48,53]. Even though no malignant transformation has been described in the literature, close follow-ups are recommended.

## Conclusion

FOPD should be considered in the differential diagnosis of fast-growing lesions of the extremities. An excisional biopsy allows for complete tumor removal and avoids overzealous treatment. FOPD should be treated without radical excisions provided if totally removed.
